# *In vivo* assessment of foveal geometry and cone photoreceptor density and spacing in children

**DOI:** 10.1038/s41598-020-65645-2

**Published:** 2020-06-02

**Authors:** Hanieh Mirhajianmoghadam, Ashutosh Jnawali, Gwen Musial, Hope M. Queener, Nimesh B. Patel, Lisa A. Ostrin, Jason Porter

**Affiliations:** 1 College of Optometry, University of Houston, 4901 Calhoun Rd, Houston, TX 77204-2020 USA; 2 Department of Biomedical Engineering, University of Houston, 3517 Cullen Blvd, Houston, TX 77204-5060 USA

**Keywords:** Image processing, Retina, Paediatric research

## Abstract

The fovea undergoes significant developmental changes from birth into adolescence. However, there is limited data examining cone photoreceptor density, foveal pit shape, and foveal avascular zone (FAZ) size in children. The purpose of this study was to determine whether overall foveal structure differs as a function of age and refractive status in children. Forty-eight healthy children (ages 5.8 to 15.8 years) underwent optical coherence tomography imaging to quantify foveal point thickness and foveal pit diameter, depth, and slope. Adaptive optics scanning laser ophthalmoscope (AOSLO) images of foveal capillaries and cone photoreceptors were acquired in a subset of children to quantify FAZ metrics and cone densities at 0.2, 0.3, and 0.5 mm eccentricities. Results show that foveal pit and FAZ metrics were not related to age, axial length, or refractive status. However, linear cone density was lower in myopic versus non-myopic children at eccentricities of 0.2 mm (mean ± SD = 50,022 ± 5,878 cones/mm^2^ vs 58,989 ± 4,822 cones/mm^2^, *P* < 0.001) and 0.3 mm (43,944 ± 5,547 cones/mm^2^ vs 48,622 ± 3,538 cones/mm^2^, *P* < 0.001). These results suggest FAZ and foveal pit metrics do not systematically differ with age in children, while myopic eyes have decreased linear cone density near the foveal center. **Significance Statement:** The development of the fovea begins prior to birth and continues through the early teenage years until it reaches adult-like properties. Although the majority of changes during childhood are related to the maturation and migration of cone photoreceptors, *in vivo* data describing cone packing in children is limited. We assessed overall foveal structure in children as young as 5.8 years old by quantifying cone density and spacing, foveal avascular zone size, and foveal pit morphometry to investigate potential structural differences as a function of age and refractive status. While foveal avascular zone and foveal pit metrics did not significantly differ with age, results indicate that myopic children have lower linear cone densities close to the foveal center compared to non-myopic children.

## Introduction

The healthy human adult fovea is a specialized retinal region that provides high spatial sampling of the retinal image^1,2^. The fovea is typically characterized anatomically by increased cone density, excavation of inner retinal layers (forming the foveal pit), and a capillary-free region known as the foveal avascular zone (FAZ). Although the location of the fovea can be identified as early as 11 weeks gestation^3^ by a single layer containing only cones^4,5^, the fovea undergoes substantial changes, both *in utero* and *ex utero*, including thinning and elongation of cone inner and outer segments^6–8^, photoreceptor migration toward the foveal center^7^, the formation of Henle’s fiber layer^6^, remodeling of the FAZ^9^, and a widening and shallowing of the foveal pit^8,10^.

Despite the formation of an identifiable fovea before birth, foveal development is a protracted process. While some aspects of the fovea are reported to mature at an early age, such as the size of the FAZ^8,9^, many studies suggest that the development and maturation of the fovea continues throughout childhood and into adolescence^8,11–14^. Age-related differences in the thicknesses of retinal layers have been broadly investigated *in vivo* in children. While the majority of changes are thought to occur within the first few years of life^6^, more recent studies report increases in the thicknesses of the outer nuclear layer^14^ and outer retinal layers in the foveal region until at least 12 years of age^13^. Reports of age-related differences in cone density are sparse. Yuodelis and Hendrickson^7^ examined 5 eyes histologically after birth and found that peak cone density at birth was only 17% of the value measured in their 37 year-old adult eye. In addition, they found that peak cone density in a 45 month-old donor eye was only half of that in the 37 year-old eye.

Few studies have quantified cone density and FAZ metrics *in vivo* in children. Park *et al*.^15^ and Tumahai *et al*.^16^ performed adaptive optics imaging of cone photoreceptors in groups of children and adults between the ages of 10–20 years and 6–20 years, respectively, at eccentricities as close as 0.5 mm and 0.45 mm from the foveal center, respectively. Despite these efforts, cone density and spacing values have yet to be described for eccentricities closer than 0.45 mm (approximately 1.5°) from the fovea in a cohort of subjects who are only children. In another study, FAZ area was quantified in 26 eyes between the ages of 10 and 19 years using optical coherence tomography angiography^17^. However, data detailing other FAZ metrics have yet to be described only in children (particularly for those under 10 years of age)^17,18^. Furthermore, there is a lack of *in vivo* data in children detailing a more comprehensive examination of overall foveal morphometry that incorporates measurements of cone density with other structural measures, such as the size of the FAZ and the foveal pit, in the same eyes.

In addition, differences in foveal cone structure are not well-described in children with different refractive errors. The prevalence of myopia continues to grow worldwide^19,20^. In the United States, approximately 60% of new myopic cases are classified as school age onset (or juvenile) myopia, typically presenting in children between 9 to 11 years of age and progressing through the early teenage years^21^. However, most of what is known about differences in cone density and foveal pit shape between myopic and non-myopic retinas comes from studies performed in fully-developed adult retinas. Cone density has been shown to be significantly lower in myopic compared to non-myopic adult eyes^22,23^, and no significant differences have been observed in the diameter, depth, and slope of the foveal pit between myopic and emmetropic subjects aged 13 to 52 years old^24^. Therefore, the main purpose of this study was to determine whether overall foveal structure, including cone density and spacing, FAZ size, and foveal pit shape, differs as a function of age and refractive status in children. This work yields a better understanding of overall foveal morphometry in children and provides insights into mechanisms of ocular growth.

## Results

Forty-eight eyes from 48 children (23 females, 25 males) were included in the study. Subject characteristics are shown in Table 1. Children had a mean age of 11.2 ± 2.7 years and ranged from 5.8–15.8 years old. Parents self-reported the race or ethnicity of their children to be White (n = 15), Hispanic (n = 19), African-American (n = 8), Asian (n = 3) and mixed (n = 3). All children were reported to be full-term (greater than 38 weeks gestation) by their parents. The mean spherical equivalent refractive error (SER) for all subjects was −0.13 ± 2.08 D (range: −5.44 D to +7.23 D). Mean axial length was 23.44 ± 1.02 mm (range: 20.86 to 25.64 mm). Eyes with longer axial lengths had more myopic spherical equivalent refractive errors (r = −0.77, *P* < 0.0001), and eyes of older children had longer axial lengths (r = 0.62, *P* < 0.0001).

**Table 1:** Demographic, refractive, and biometric characteristics for all 48 children (All Subjects) and separated by refractive group (Non-myopes, Myopes). Data are presented as mean ± standard deviation (range).

	All Subjects	Non-myopes (SER > −0.50 D)	Myopes (SER ≤ −0.50 D)	P value
Number of subjects	48	30	18	—
Sex (Female/Male)	23 / 25	16 / 14	7 / 11	—
Age (years)	11.2 ± 2.7 (5.8 to 15.8)	10.2 ± 2.6 (5.8 to 15.8)	12.9 ± 1.9 (8.5 to 15.8)	<0.001*
Spherical Equivalent Refractive Error (D)	−0.13 ± 2.08 (−5.44 to +7.23)	0.96 ± 1.64 (−0.44 to +7.23)	−1.96 ± 1.32 (−5.44 to −0.56)	<0.001*
Axial length (mm)	23.44 ± 1.02 (20.86 to 25.64)	22.94 ± 0.84 (20.86 to 24.54)	24.26 ± 0.72 (22.81 to 25.64)	<0.001*
Anterior corneal radius of curvature (mm)	7.75 ± 0.24 (7.29 to 8.36)	7.79 ± 0.24 (7.42 to 8.36)	7.68 ± 0.22 (7.29 to 8.19)	0.1
Corneal thickness (µm)	557.9 ± 30.0 (487 to 633)	565.0 ± 29.7 (492 to 633)	545.9 ± 27.2 (487 to 598)	0.03*
Anterior chamber depth (mm)	3.74 ± 0.30 (2.96 to 4.38)	3.64 ± 0.28 (2.96 to 4.19)	3.92 ± 0.26 (3.54 to 4.38)	<0.01*
Lens thickness (mm)	3.43 ± 0.23 (3.05 to 4.55)	3.48 ± 0.25 (3.12 to 4.55)	3.34 ± 0.18 (3.05 to 3.79)	0.04*

*Indicates a statistically significant difference between non-myopes and myopes (*P* < 0.05).

### Foveal parameters with age and axial length

Mean foveal pit metrics for all subjects (n = 48) are shown in Table 2. Across subjects, the range of foveal pit metrics varied between a factor of 1.3 (for foveal point thickness) and 2.2 (for foveal pit slope). Because age and axial length were found to be correlated, we performed a multiple linear regression analysis (with age and axial length as independent variables) to determine the extent to which each foveal pit metric was related to age and axial length. We found that no foveal pit metrics were significantly correlated with age or axial length (Table 2).

**Table 2:** Mean (±standard deviation) foveal pit and foveal avascular zone (FAZ) parameters, and their correlation with age and axial length across all children.

	Mean ± SD (Range)	R^2^	Axial length (P Value)	Age (P Value)
**Foveal Pit Metrics (n = 48)**				
Foveal Pit Depth (µm)	129 ± 15 (99 to 170)	0.03	0.23	0.64
Foveal Pit Diameter (mm)	2.16 ± 0.19 (1.67 to 2.61)	0.06	0.10	0.33
Foveal Pit Slope (degrees)	16.5 ± 3.1 (11.5 to 25.6)	0.11	0.61	0.15
Foveal Point Thickness (µm)	220 ± 13 (195 to 258)	0.03	0.49	0.25
**FAZ Metrics (n = 31)**				
FAZ Area (mm^2^)	0.302 ± 0.125 (0.051 to 0.557)	0.10	0.21	0.61
FAZ Perimeter (mm)	2.503 ± 0.666 (1.009 to 3.853)	0.04	0.93	0.35
Axis Ratio (unitless)	1.18 ± 0.15 (1.01 to 1.63)	0.04	0.31	0.96
FAZ Effective Diameter (µm)	606 ± 138 (255 to 842)	0.09	0.21	0.79
Circularity (unitless)	0.60 ± 0.15 (0.38 to 0.88)	0.12	0.06	0.27
Acircularity (unitless)	1.32 ± 0.16 (1.07 to 1.63)	0.11	0.07	0.27

Mean FAZ parameters for all analyzed subjects (n = 31) are also shown in Table 2. FAZ parameters and cone metrics were analyzed for a subset of subjects as not all parents provided permission for adaptive optics scanning laser ophthalmoscope (AOSLO) imaging, and some children found it challenging to maintain adequate fixation during imaging. FAZ area showed considerable variability between subjects, with an approximately 11-fold range of values (between 0.051 to 0.557 mm^2^). After performing a similar multiple linear regression analysis (with age and axial length as independent variables), we found that no FAZ metrics were significantly correlated with age or axial length. However, there was a tendency for FAZ circularity to increase (*P* = 0.06) and FAZ acircularity to decrease (*P* = 0.07) with increasing axial length.

**Figure 1:**
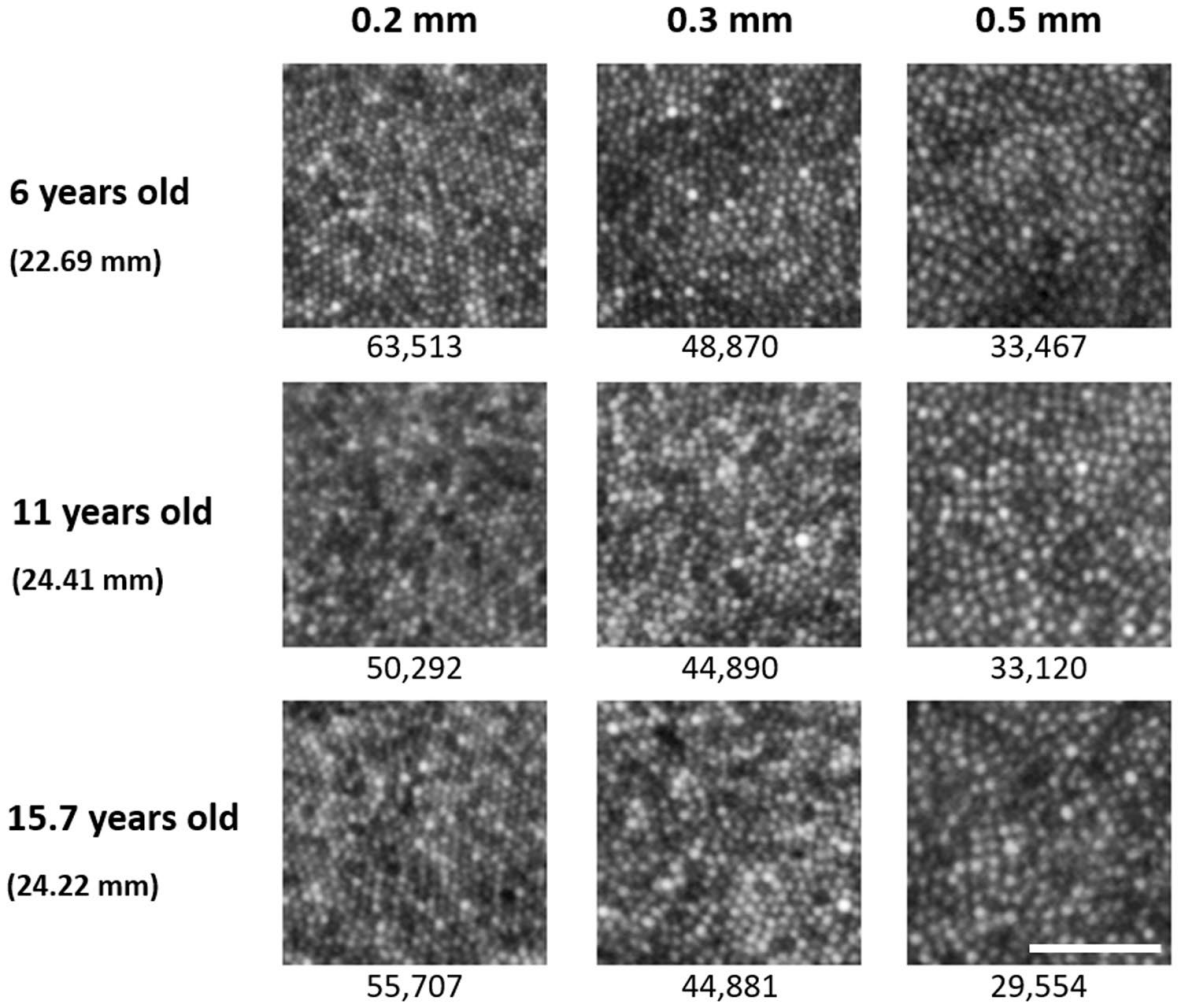
Representative cone photoreceptor images acquired along the inferior meridian in three subjects at the three examined eccentricities of 0.2 mm, 0.3 mm, and 0.5 mm from the estimated location of peak cone density. Each subject’s axial length is indicated in parentheses beneath their age. Linear cone density (cones/mm^2^) is specified for each image. Linear cone photoreceptor density decreases with increasing eccentricity in each eye. Scale bar = 50 μm.

**Figure 2:**
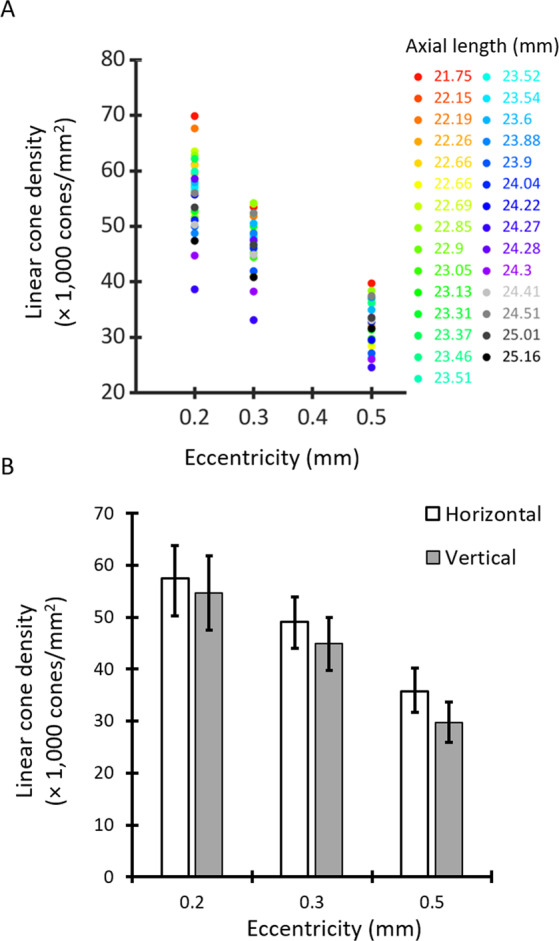
(**A**) Linear cone densities (cones/mm^2^) averaged across all meridians for the 29 children examined at the three retinal eccentricities. Each subject is represented by a different color. (**B**) Mean linear cone densities averaged across horizontal (no fill) and vertical (gray) meridians. Error bars represent ±1 standard deviation. Linear cone densities were significantly higher along the horizontal meridian than the vertical meridian across all eccentricities.

Montages of the foveal cone mosaic were created for one eye of each of 29 children. Representative images of the cone mosaic are shown in Fig. 1 for three subjects of different ages at each of the three examined eccentricities. Linear cone densities for all subjects are plotted at the eccentricities examined in Fig. 2A. Mean linear cone densities across all children and meridians were 55,897 ± 6,698 cones/mm^2^, 47,009 ± 4,802 cones/mm^2^, and 32,556 ± 3,954 cones/mm^2^ at 0.2 mm, 0.3 mm, and 0.5 mm eccentricities, respectively. A one-way ANOVA and Bonferroni post-hoc test revealed that linear and angular cone densities were significantly higher at an eccentricity of 0.2 mm compared to 0.3 mm (*P* < 0.001), and at an eccentricity of 0.3 mm compared to 0.5 mm (*P* < 0.001). A comparison of our mean linear cone density values with other histological and *in vivo* studies is shown in Table 3. Figure 2B shows mean linear cone densities along the horizontal and vertical meridians across the 29 examined subjects.

**Table 3:** Comparison of linear cone density measurements between previously published *ex vivo* and *in vivo* studies with measurements from the current study.

Study	*Ex Vivo*	*In Vivo* Adult				*In Vivo* Children/Adult	*In Vivo* Children
	Curcio *et al*.^25^	Li *et al*.^26^	Song *et al*.^30^	Elsner *et al*.^27^	Lombardo *et al*.^23^	Park *et al*.^15^	Current work
Number of subjects	7	18	10	36	12	36	29
Age range (years)	27–44	23–44	22–35	18–34	24–38	10–20	5–16
Axial length range (mm)	Not reported	22.9–28.3	22.1–26.1	23.2–25.8	22.6–26.6	22.01–28.43	20.86–25.64
**Linear Cone Density (cones/mm**^**2**^**) at eccentricities of:**							
~0.20 mm	73,000	78,900 ± 9300	67,400 ± 15,600 (0.18 mm)	Not reported	Not reported	Not reported	55,897 ± 6,698
~0.30 mm	53,500	57,000 ± 6800	56,700 ± 7,900 (0.27 mm)	43,216 ± 6,039 (0.27 mm)	49,393 ± 7,941 (0.26 mm)	Not reported	47,009 ± 4,802
~0.40 mm	42,200	Not reported	46,800 ± 8,300 (0.36 mm)	Not reported	38,000	Not reported	Not reported
≥0.50 mm	36,000 (0.50 mm)	Not reported	38,800 ± 7,500 (0.45 mm)	27,466 ± 3,496 (0.63 mm)	30,049 ± 4,954 (0.6 mm)	32,554 ± 2,884 (0.50 mm)	32,556 ± 3,954 (0.50 mm)
Meridians reported	All	All	All	All	Horizontal	All	All

**Figure 3:**
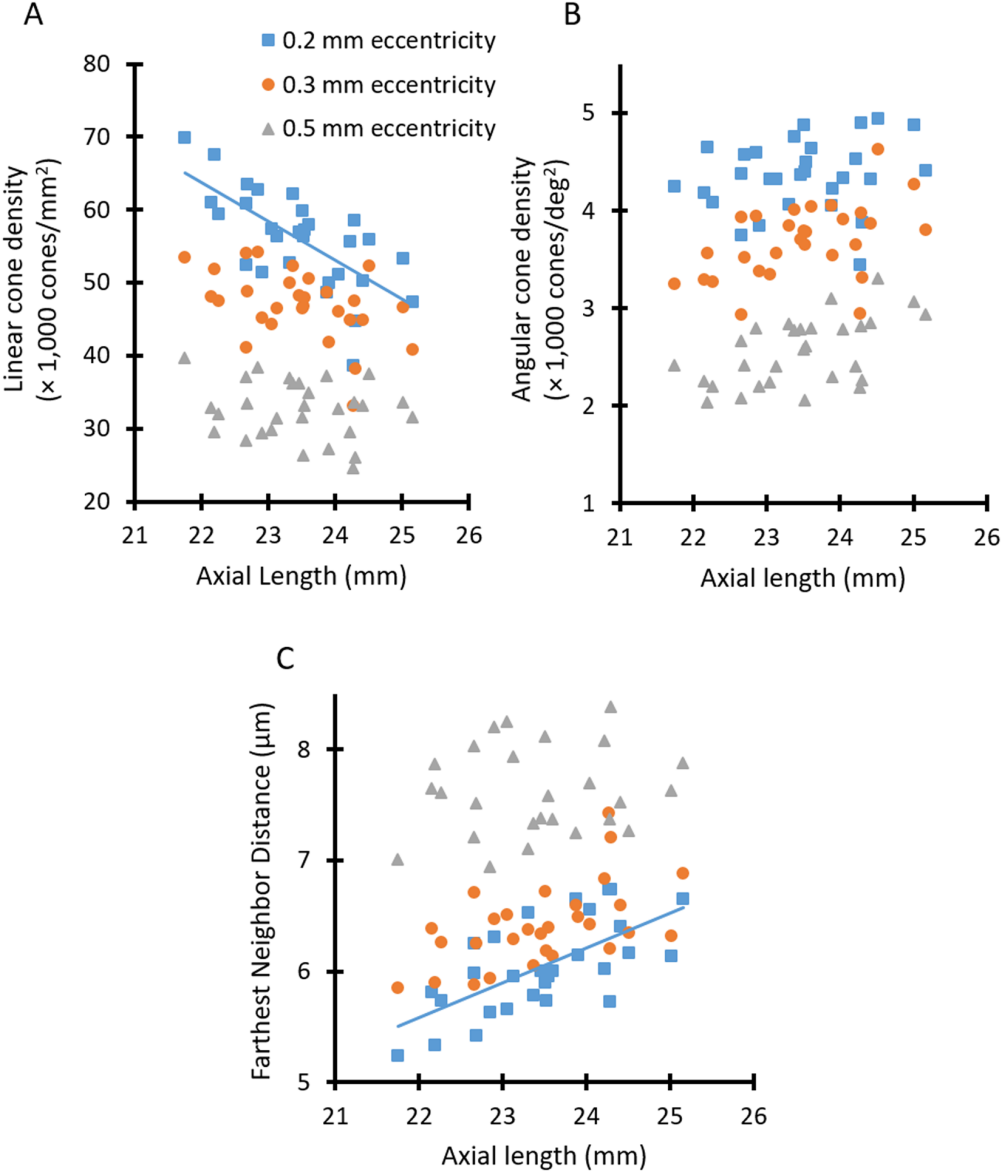
Cone metrics were analyzed as a function of axial length at eccentricities of 0.2 mm (blue squares), 0.3 mm (orange circles), and 0.5 mm (gray triangles) for 29 subjects. Lines show linear regressions for those data that possessed a statistically significant relationship. (**A**) Linear cone density (cones/mm^2^) significantly decreased with increasing axial length only at an eccentricity of 0.2 mm (linear cone density [x 1,000 cones/mm^2^] = −5.2913 × (axial length, in mm) +180.08, *P* = 0.001). (**B**) Angular cone density (cones/deg^2^) was not significantly related to axial length at any examined eccentricity. (**C**) Farthest neighbor distance significantly increased in eyes with longer axial lengths only at an eccentricity of 0.2 mm (farthest neighbor distance = 0.312 × (axial length, in mm) − 1.2785, *P* = 0.002).

Cone metrics as a function of axial length for three different retinal eccentricities are shown in Fig. 3. A multiple linear regression was performed for each cone metric measured at each eccentricity, with age and axial length as independent variables, and the cone metric as the dependent variable. These analyses yielded statistically significant relationships only for metrics analyzed at an eccentricity of 0.2 mm. At this eccentricity, the model explained 50.4% of the variance in linear cone density (cones/mm^2^), and revealed that linear cone density significantly decreased with increasing axial length (*P* = 0.001, Fig. 3A). Similarly, 46.8% of the variance in farthest neighbor distance (when measured at an eccentricity of 0.2 mm) could be explained using a multiple linear regression model. The model showed that eyes with longer axial lengths also had increased cone spacing (i.e., increased farthest neighbor distance) (*P* = 0.002, Fig. 3C). Neither axial length nor age contributed to the regression models for angular cone density (cones/deg^2^) at any eccentricity (Fig. 3B).

### Foveal parameters between myopic and non-myopic children

Foveal pit and FAZ metrics were compared between eyes of children that were myopic (SER ≤ −0.50 D) and non-myopic (SER > −0.50 D) (Table 4). There were no significant differences in foveal pit metrics between refractive error groups. However, myopic eyes tended to have flatter foveal pits compared to non-myopes (foveal pit slope = 15.5 ± 2.5° vs. 17.2 ± 3.3°, respectively, *P* = 0.06). In addition, no statistically significant differences in FAZ metrics were found between the two refractive error groups (*P* > 0.05).

**Table 4:** Mean (±standard deviation) foveal pit and FAZ metrics in myopic and non-myopic children.

	Myopic	Non-myopic	P Value
**Foveal Pit Metrics (n = 48)**			
Number of Subjects	18	30	—
Foveal Pit Depth (µm)	129 ± 16	129 ± 14	0.83
Foveal Pit Diameter (mm)	2.21 ± 0.21	2.13 ± 0.18	0.18
Foveal Pit Slope (degrees)	15.5 ± 2.5	17.2 ± 3.3	0.06
Foveal Point Thickness (µm)	220 ± 16	219 ± 10	0.86
**FAZ Metrics (n = 31)**			
Number of Subjects	11	20	—
FAZ Area (mm^2^)	0.338 ± 0.162	0.283 ± 0.099	0.25
FAZ Perimeter (mm)	2.592 ± 0.805	2.454 ± 0.593	0.59
Axis Ratio	1.14 ± 0.08	1.21 ± 0.17	0.24
FAZ Effective Diameter (µm)	632 ± 185	591 ± 107	0.44
Circularity	0.61 ± 0.15	0.60 ± 0.15	0.88
Acircularity	1.31 ± 0.17	1.32 ± 0.17	0.88

**Figure 4:**
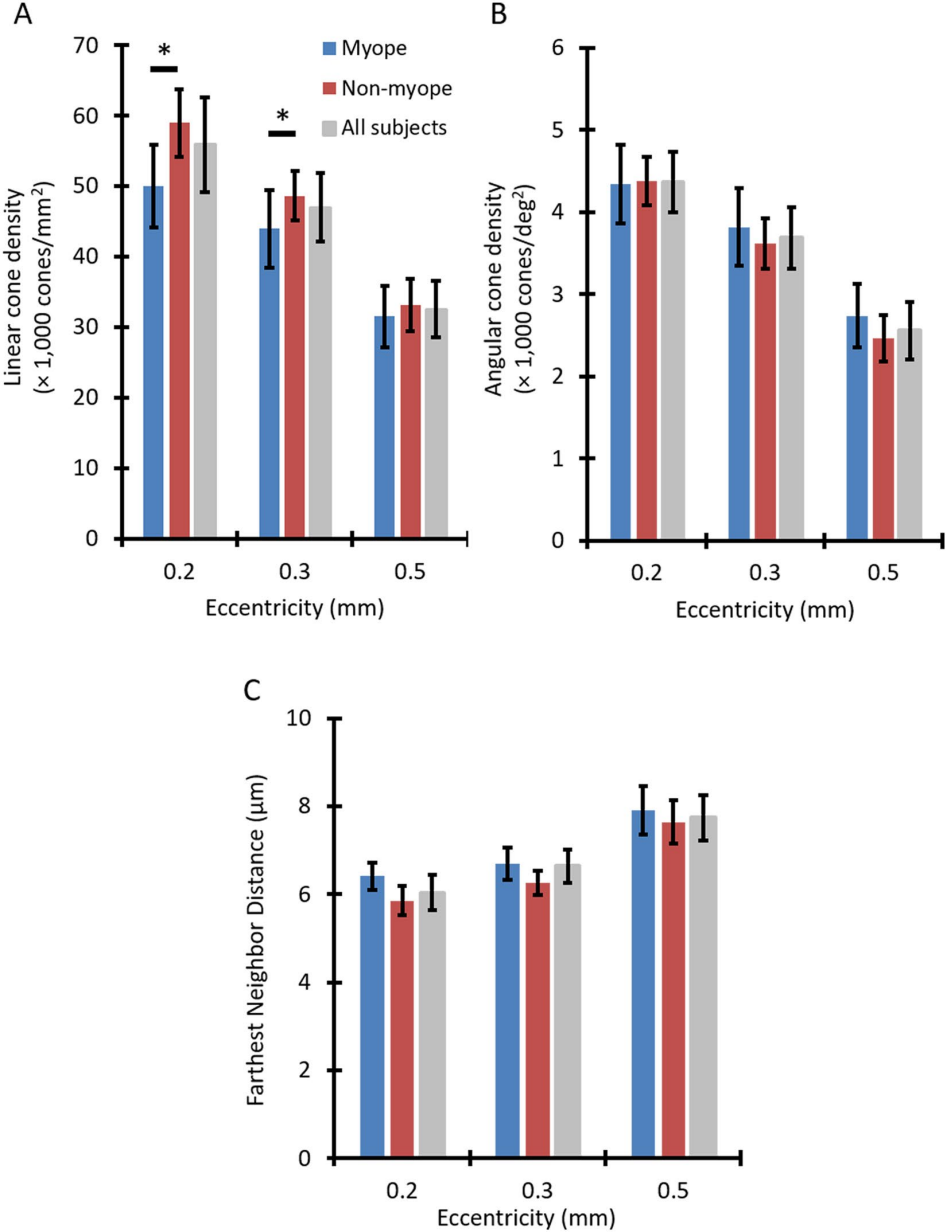
Differences in cone density and spacing metrics were found between myopic and non-myopic groups. Mean (**A**) linear cone density, (**B**) angular cone density, and (**C**) farthest neighbor distance averaged across 10 myopic children (blue), 19 non-myopic children (red), and all 29 children combined (gray) as a function of eccentricity. Error bars represent ±1 standard deviation. Asterisks indicate a statistically significant difference between myopic and non-myopic groups at a given retinal eccentricity (*P* < 0.001). (**A**) Linear cone density was significantly lower in myopic children at 0.2 and 0.3 mm eccentricities. (**B**) Angular cone density was significantly higher across all eccentricities combined in myopic children. (**C**) Farthest neighbor distance was also significantly higher across all eccentricities combined in myopic children.

Mean values of all three cone metrics are presented in Fig. 4 for myopic and non-myopic groups at the examined eccentricities. At an eccentricity of 0.2 mm, mean linear cone density was 50,022 ± 5,878 cones/mm^2^ for the myopic group versus 58,989 ± 4,822 cones/mm^2^ for the non-myopic group (Fig. 4A). At a retinal eccentricity of 0.3 mm, mean linear cone densities for myopic and non-myopic groups were 43,944 ± 5,547 cones/mm^2^ and 48,622 ± 3,538 cones/mm^2^, respectively. The difference in linear cone density between refractive groups was lowest at 0.5 mm eccentricity (31,512 ± 4,389 cones/mm^2^ for myopes vs 33,105 ± 3,710 cones/mm^2^ for non-myopes).

Table 5 presents results from three-way ANOVAs that were performed to investigate whether there were any significant effects of eccentricity, meridian, and refractive error group on cone density and spacing parameters, and whether there were significant interactions between these variables. For both linear and angular cone densities, significant effects of eccentricity, meridian, and refractive group were observed (*P* < 0.05 for each variable). Linear and angular cone densities were significantly higher along the horizontal meridian than the vertical meridian (*P* < 0.001 for both, Fig. 2B). A significant interaction was observed between eccentricity and refractive group (*P* < 0.001) for linear cone density, with myopic children showing decreased linear cone density compared to non-myopic children at eccentricities of 0.2 mm and 0.3 mm (*P* < 0.001). Similarly, for farthest neighbor distance, significant effects of eccentricity, meridian, and refractive status were observed, with a significant interaction between eccentricity and meridian (*P* < 0.001 for all). Farthest neighbor distance was greater for myopic versus non-myopic children across all eccentricities combined (*P* < 0.001), and was smaller in the horizontal meridian versus the vertical meridian at an eccentricity of 0.5 mm (*P* < 0.001).

**Table 5:** Main effects and interaction effects of eccentricity, meridian, and refractive group on cone photoreceptor density and spacing metrics.

	P value		
	Linear Cone Density (cones/mm^2^)	Angular Cone Density (cones/deg^2^)	Farthest Neighbor Distance
Main effect of eccentricity	<0.001*	<0.001*	<0.001*
Main effect of meridian	<0.001*	<0.001*	<0.001*
Main effect of refractive group	<0.001*	0.011*	<0.001*
Interaction effect of eccentricity x meridian	0.185	0.151	<0.001*
Interaction effect of eccentricity x refractive group	<0.001*	0.125	0.231
Interaction effect of meridian x refractive group	0.672	0.360	0.244
Interaction effect of eccentricity x meridian x refractive group	0.662	0.601	0.278

*P < 0.05 was regarded as statistically significant.

In order to provide a more comprehensive assessment of the foveal region in children, we examined whether relationships exist between various metrics (including FAZ and foveal pit parameters) across all eyes. Eyes with larger FAZ areas had wider and deeper foveal pits (r = 0.65, *P* < 0.001, and r = 0.56, *P* = 0.002, respectively). FAZ area was also smaller in eyes with greater foveal point thicknesses (r = −0.70, *P* < 0.001), while eyes with thinner foveal point thicknesses had deeper foveal pits (r = −0.61, *P* = 0.001). Linear cone density at an eccentricity of 0.2 mm was lower in eyes with larger FAZ areas (r = −0.45, *P* = 0.02) and wider foveal pits (r = −0.40, *P* = 0.03).

## Discussion

The main purpose of this study was to determine whether overall foveal structure differs as a function of age and refractive status in children. Eyes with myopic refraction and increased axial length tended to have decreased linear cone density close to the fovea. Linear cone density (cones/mm^2^) was significantly lower in eyes of myopic children compared to non-myopic children at 0.2 and 0.3 mm eccentricities. However, foveal avascular zone and foveal pit geometry were independent of age, axial length, and refractive status.

None of the foveal avascular zone, foveal pit, or cone density metrics measured in the children in this study showed a significant relationship with age, suggesting that the foveal parameters investigated here may be close to being fully developed. Although studies have shown that foveal development is a protracted process^8,11^, the rate of development and maturation of different aspects of the fovea is not constant throughout infancy, childhood, and adolescence. For example, Lee *et al*.^14^ reported that outer nuclear layer, inner segment, and outer segment thicknesses increase logarithmically between birth and the first few years after birth, after which the thicknesses of these layers increase more gradually during childhood and adolescence. An increase in the thickness of the outer nuclear layer could be explained by the migration of cone photoreceptors toward the foveal center. However, there is no direct evidence in the literature as to when cones cease centripetal migration or the rate at which they migrate over time. Based on Yuodelis and Hendrickson’s work^7^, cone density for a 37 year-old donor eye was twice the density of a 45 month-old donor eye, whose density was ten times greater than the density of a donor eye at 22 weeks gestation.

This study is the first to report *in vivo* cone densities specifically in young children at retinal eccentricities within 0.45 mm (~1.5°) of the foveal center. Published cone photoreceptor data in children under 18 years of age are sparse. Park *et al*.^15^ used AOSLO imaging to measure cone densities at 0.5, 1.0, and 1.5 mm eccentricities in subjects between 10 and 20 years old. More recently, Tumahai *et al*.^16^ used an adaptive optics retinal camera (RTX-1, Imagine Eyes) to measure cone density at an eccentricity of 0.45 mm in the nasal and temporal meridians of subjects between 6 and 20 years of age. In both of these studies, children were not classified separately from adults and mean cone density was reported for all subjects younger than 20 years old, making it challenging to determine potential differences in density between children and adult eyes. Building on these reports, we detail cone density measurements in children as young as 5.8 years old (range: 5.8–15.8 years) at eccentricities as close as 0.2 mm from the fovea.

Linear cone density values measured in the eyes of the children in our study are comparable to those measured in adolescent eyes at slightly more peripheral locations (0.5 mm) (Table 3). The mean value of linear cone density calculated in the children in this study at an eccentricity of 0.5 mm (32,556 ± 3,954 cones/mm^2^) was similar to the values reported by Park *et al*.^15^ in 10 to 20 year old subjects at an eccentricity of 0.5 mm (32,554 ± 2,884 cones/mm^2^) and by Tumahai *et al*.^16^ in 6 to 20 year old subjects at an eccentricity of 0.45 mm (~36,500 cones/mm^2^). However, given the lack of published data on cone density in children at closer eccentricities, we also compared measurements from the children in our study with those from studies performed in adult eyes. While the cone density values reported in this study are lower than *ex vivo* values reported by Curcio *et al*.^25^ and *in vivo* values reported by Li *et al*.^26^, they are within the range of reported *in vivo* values in adult eyes (Table 3). For example, at an eccentricity of 0.3 mm, the mean linear cone density measured in this study (47,009 ± 4,802 cones/mm^2^) is less than the mean value reported by Li *et al*.^26^ (57,000 ± 6800 cones/mm^2^) at 0.3 mm in 18 adult eyes, but is greater than the mean values reported by Elsner *et al*.^27^ (43,216 ± 6,039 cones/mm^2^) at 0.27 mm in 36 adult eyes (where density would be expected to be greater than that measured at 0.3 mm). Similarly, at an eccentricity of 0.2 mm, the mean linear cone density measured in this study (55,897 ± 6,698 cones/mm^2^) is lower than the values reported by Curcio *et al*.^25^ and Li *et al*.^26^ (73,000 and 78,900 ± 9,300 cones/mm^2^, respectively). However, our mean value of cone density along the horizontal meridian at the same eccentricity of 0.2 mm (57,412 ± 6,427 cones/mm^2^) is similar to values reported by Lombardo *et al*.^28^ at an eccentricity of 0.25 mm along the horizontal meridian (mean = 57,508 cones/mm^2^). Even though Hendrickson *et al*.^8^ found that the thickness of the outer nuclear layer increases from one nucleus thick at birth to 12 nuclei-thick at 13 years of age, we did not observe a significant relationship between cone density and age after controlling for the effect of axial length. In addition, the cone density values measured in the children in our study are already within an adult-like range. Hence, it is possible that the majority of cone photoreceptor migration toward the foveal center occurs by a younger age and/or cone migration beyond the age of 5 occurs at a rate that is slower compared to first few years of life. Further study is warranted in children younger than 5 years of age, as well as in longitudinal studies, to elucidate differences in cone density during infancy and childhood. Such studies could additionally provide improved understanding of the variability in cone density and spacing metrics for children of a given age, as well as across ages.

Consistent with results reported histologically^25^ and *in vivo* in adult eyes^15,29,30^, we observed higher linear cone densities along the horizontal meridian in children (Fig. 2B). Differences in cone density were measured between the horizontal and vertical meridians across all eccentricities. However, the difference was statistically significant only for farthest neighbor distance at an eccentricity of 0.5 mm.

No relationships were found between foveal pit metrics and age in the children in our study. Previous histological studies performed in a limited set of retinas from children have reported that the foveal pit becomes wider and shallower postnatally^8,10^. More recently, Read *et al*.^13^ performed *in vivo* OCT imaging in nearly 200 children between 4 and 12 years of age and found a significant increase in foveal thickness with age. However, we did not find any relationships between foveal pit shape or foveal point thickness and age in our cohort of 5 to 15 year old children. These conflicting results may stem from multiple sources, including differences in age, race, and refractive error between studies. For example, subjects in the Read *et al*.^13^ study were younger in age relative to our subjects, and 90% of their sample was White, whereas our population was more diverse (primarily consisting of 31% White, 40% Hispanic, and 17% African-American). Moreover, the range of spherical equivalent refractive errors in the Read *et al*.^13^ study was close to emmetropia (−0.50 D to +1.25 D), while the spherical equivalent refractive errors in our sample were more broadly distributed, ranging from −5.44 D to +7.23 D.

Values of foveal pit metrics measured in our cohort are similar to those reported in previous studies in children and adults. Mean (± standard deviation) values of foveal pit depth and slope in our study (129 ± 15 µm and 16.5 ± 3.1degrees, respectively) are comparable to values reported by Yanni *et al*.^18^ in 34 normal children aged 5 to 16 years (129.6 ± 35.6 µm and 14.6 ± 5.2 degrees, respectively), while foveal pit diameter was slightly larger in our group (2.16 ± 0.19 mm vs 1.77 ± 0.35 mm). Foveal pit metrics in the Yanni *et al*.^18^ study were quantified from a single horizontal (and vertical) line scan bisecting the fovea, which ignores the heterogeneity of foveal pit structure measured along different meridians. In our study, we built on the approach introduced by Dubis *et al*.^24^ to model the foveal pit in three dimensions, thereby better representing the overall shape of the pit and providing more accurate measures of pit parameters. In addition to being similar to previous reports in children, the range of values for foveal pit depth (99–170 µm), diameter (1.67–2.61 mm), and slope (11.5–25.6 degrees) in the current study are similar, but slightly higher, than those reported in adults by Dubis *et al*.^31^ (48–156 µm, 1.12–2.40 mm, and 5.1–21 degrees, respectively).

Linear cone density significantly decreased in eyes with longer axial lengths only at an eccentricity of 0.2 mm (Fig. 3A), indicating that axial elongation impacts cone density at retinal locations closer to the foveal center. We were unable to image the central-most foveal cones in any children, and therefore could not determine whether this trend was also true at the foveal center. However, when coupled with no observed change in angular cone density with increasing axial length, this decrease in linear cone density at 0.2 mm eccentricity supports a simple scaling model of growth in which the size and spacing of the more central cones increase in response to axial elongation. It would be valuable to measure cone densities at locations closer to the foveal center and at more peripheral eccentricities to provide more information on whether axial elongation differentially impacts retinal structure at different eccentricities.

In agreement with previous studies in adults, linear cone density is significantly lower in myopic compared to non-myopic children^22,23^. Specifically, the eyes of myopic children had significantly lower linear cone densities compared to the eyes of non-myopic children at retinal eccentricities of 0.2 mm and 0.3 mm (three-way ANOVA, post-hoc comparison, *P* < 0.001). Lombardo *et al*.^23^ examined linear cone density in young adult eyes at eccentricities of 0.26, 0.4, and 0.6 mm and found that the mean difference in cone density between emmetropic and myopic eyes was greatest at an eccentricity of 0.26 mm (7,709 cones/mm^2^) and lowest at an eccentricity of 0.6 mm (4,536 cones/mm^2^). Likewise, in our study, the mean difference in linear cone density was highest at the closest eccentricity examined (8,967 cones/mm^2^ at 0.2 mm eccentricity) and lowest at the furthest eccentricity examined (1,593 cones/mm^2^ at 0.5 mm eccentricity).

We found no significant correlations between any foveal pit metric and axial length in our cohort. Axial length has been shown to dramatically increase during the first few years of life^32^, and retinal stretching (potentially resulting from axial elongation) has been proposed as a mechanism for altering the morphometry of the foveal pit^10^. It is possible that the lack of relationship found between axial length and foveal pit metrics in our study could have been confounded by the racial makeup of our subjects. Wagner-Schuman *et al*.^33^ reported that differences in foveal pit architecture exist between White and African-American adult subjects. Given the more heterogeneous ethnic composition of our cohort, future studies could include additional children across different racial and ethnic groups. In addition, we found no significant difference in foveal pit morphometry between myopic and non-myopic children. This finding is consistent with results reported by Dubis *et al*.^24^ in 61 healthy emmetropic and myopic subjects aged between 13 and 52 years.

None of the FAZ metrics calculated in this study were correlated with age or axial length. Previous work conducted by Cheung *et al*.^34^ in a population of children aged 6–8 years old in Hong Kong found that FAZ area decreases in eyes with longer axial lengths. The discrepancy between their findings and those reported in this study could be due to several factors, including differences in the range of ages and ethnicities of both groups. Furthermore, Cheung *et al*.^34^ did not report whether they laterally scaled the magnification of their retinal images to correct for differences in axial length between subjects, providing another potential source for the discrepancy. In alignment with our results in children, studies conducted in adult eyes which have scaled for retinal magnification have reported no significant relationship between FAZ area and axial length^17,31^. In addition, the mean value and range of FAZ area measured in the children in this study (mean = 0.302 ± 0.125 mm^2^; range: 0.051–0.557 mm^2^) are comparable to those recently measured in children between the ages of 9 weeks to 17 years by Hsu *et al*.^35^ (mean = 0.347 ± 0.168 mm^2^; range: 0.05–1.17 mm^2^) and 9–18 years by Golebiewska *et al*.^36^ (range: 0.004–0.563 mm^2^). The range of FAZ areas measured in our study is also in agreement with the range of FAZ areas imaged using adaptive optics in adult eyes^37^.

In conclusion, this study provides a comprehensive, *in vivo* assessment of foveal morphometry and cone density in healthy children. Our work expands upon previously published studies on retinal structure of children in several ways. First, this study quantifies cone density and spacing in a group of children as young as 5.8 years of age at eccentricities as close as 0.2 mm from the fovea. Second, we investigated the size of the FAZ, morphometry of foveal pit, and cone density and spacing in the same subjects to provide a more comprehensive assessment of the foveal region in children. We found that age was not correlated with any foveal pit, FAZ, or cone density or spacing metric. FAZ and foveal pit morphometry were not different between myopic and non-myopic children whereas linear cone density was lower in myopic children at 0.2 and 0.3 mm eccentricities compared to non-myopic children.

## Methods

Healthy children, 5.8 to 15.8 years of age, were recruited to participate in this study. After explaining the nature of the study, informed consent for study participation was acquired in the form of written informed assent from all children and written permission from all parents. The study followed the tenets of the Declaration of Helsinki and was approved by the University of Houston Institutional Review Board.

Children underwent a comprehensive vision examination to determine ocular refraction, best corrected visual acuity, biometry, and ocular health status. All children had a best corrected visual acuity of 20/20 or better. Both eyes were dilated using 1% tropicamide (Henry Schein Inc., Melville, NY USA) and 2.5% phenylephrine (Paragon BioTeck Inc., Portland, OR USA). Upon dilation, autorefraction was performed (WAM-5500, Grand-Seiko, Hiroshima, Japan); at least five measurements were taken and averaged for each eye. The refractive status for each child was determined from the average spherical equivalent refractive error (SER) of the examined eye, and was classified as myopic (cycloplegic SER ≤ −0.50 D) or non-myopic (cycloplegic SER > −0.5 D).

### Ocular biometry and image scaling

Axial length, anterior corneal radius of curvature, corneal thickness, anterior chamber depth, and lens thickness were measured in each eye using an ocular biometer (LenStar LS 900, Haag-Streit, Koeniz, Switzerland). Biometry data were used to laterally scale adaptive optics scanning laser ophthalmoscope (AOSLO) and spectral domain optical coherence tomography (SDOCT) images using a four surface eye model. The refractive index of the cornea was taken to be 1.38^38^, and the posterior radius of curvature of the cornea was modeled after Williams^39^ to be 0.8831 ×(anterior radius of curvature of the cornea). The schematic eye model was modified for children by incorporating previously published values of the anterior and posterior radii of curvatures of the lens based on the age of each child using the following equations:^40^

1 $$lens\,anterior\,radius\,of\,curvature\,(mm)=-\,0.021{x}^{2}+0.151x+11.45$$

2 $$lens\,posterior\,radius\,of\,curvature\,(mm)=0.004{x}^{2}+0.063x+6.236$$

where x is the (age in years – 10). The refractive indices for the aqueous, lens, and vitreous were taken from LeGrand’s Complete Theoretical eye^41^.

### Spectral domain optical coherence tomography (SDOCT) imaging & analysis

**Figure 5:**
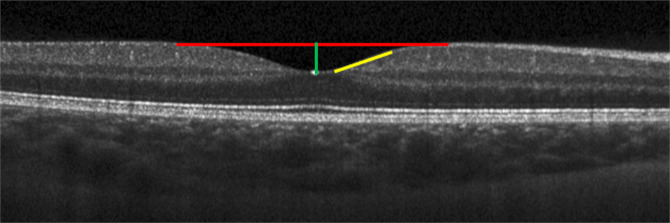
Representative SDOCT B-scan acquired through the center of the fovea and used to calculate foveal pit metrics, including foveal pit diameter (red line), foveal pit depth (green line), and foveal pit slope (yellow line).

High resolution volume scans centered on the macula (97 B-scans, 1024 A-scans/B-scan) were acquired over a 20° × 20° field in the right eye of each child using spectral domain optical coherence tomography (SDOCT; Spectralis HRA+OCT, Heidelberg Engineering, Heidelberg, Germany) and used to calculate foveal pit parameters (Fig. 5). SDOCT and adaptive optics scanning laser ophthalmoscope (AOSLO) images could not be acquired with sufficient quality in the right eye of 4 subjects due to poor fixation. Therefore, images were acquired from the left eyes of these subjects for both modalities and used for the study.

The internal limiting membrane and Bruch’s membrane were automatically delineated in each SDOCT B-scan using the instrument’s onboard segmentation algorithm and confirmed by visual inspection. Segmented B-scans were imported into a custom MATLAB program (Mathworks, Inc., Natick, MA USA) to generate retinal thickness maps, from which 24 radially oriented slices (each separated by 15°) were interpolated through the center of the fovea and smoothed. For each radial slice, the difference in retinal thickness between adjacent points along the smoothed profile (i.e. the numerical derivative) was calculated to determine the slope of the foveal contour. Locations with a numerical derivative of zero corresponded to the peaks of the foveal rim and the bottom of the foveal pit within each radial slice. The pit diameter for a single radial slice was defined as the distance between the peak locations of the foveal rim on both sides of the pit (Fig. 5). Mean foveal pit diameter for each eye was calculated as the average pit diameter across all 24 radial slices. Foveal pit depth within each slice was calculated as the difference between the mean retinal thickness of both peak rim locations and the retinal thickness at the bottom of the foveal pit. Mean foveal pit depth for each eye was calculated as the average pit depth across all 24 radial slices. Mean foveal pit slope was calculated for each eye as the average of the maximum slopes between the foveal rims and the bottom of the pit across all radial scans. Using the retinal thickness maps for each eye, we identified locations along the foveal contour within the pit that had heights corresponding to 10–70% of the mean foveal pit depth in 2% increments. For each interval, an ellipse was fit to the points with the same percent height. The centers of the 30 fitted ellipses were averaged and used to determine the mean center of the foveal pit. Foveal point thickness^13^ was calculated as the retinal thickness at the mean center of the foveal pit.

### Adaptive optics scanning laser ophthalmoscope (AOSLO) imaging & analysis

Cone photoreceptors and the foveal avascular zone (FAZ) were also imaged in a subset of children using an AOSLO^42^. A custom bite bar was made for each child and attached to a 3-dimensional translation stage to align the child’s pupil with the optical axis of the instrument and to minimize head movements during image acquisition. Children were instructed to view a digitally controlled fixation target presented at different locations within the AOSLO system. The fixation light was moved from the foveal center (0°) to a retinal eccentricity of 2° in 0.5° steps in multiple directions to acquire images over a roughly 4° × 4° patch of retina, centered on the fovea. At each retinal location, aberrations were measured over a dilated pupil (approximately 8 mm) at a wavelength of 840-nm (S Series Broadlighter Superluminescent Diode, S-840-B-I-20, Superlum, Carrigtwohill, Ireland; Full Width at Half Maximum = 50 nm) using a Shack-Hartmann wavefront sensor and were corrected using a deformable mirror (Hi-Speed DM97–15, ALPAO, Montbonnot-Saint-Martin, France). Light levels for aberration correction and imaging (peak power of ~300 μW at the corneal plane) were more than 10 times below the maximum permissible exposure established by the ANSI standards^43,44^.

Confocal and split detector videos of cone photoreceptors and retinal vasculature comprising the FAZ were simultaneously collected over a 1° or 1.5° field of view at a rate of 25 Hz at each retinal location. After correcting for scan distortions, a strip-registration technique was used to remove inter- and intra-frame eye motion, and create a stabilized video and registered image for each confocal video^45,46^. The same offsets were then applied to identical strips from each frame in the corresponding split detector video to generate stabilized split detector videos.

**Figure 6:**
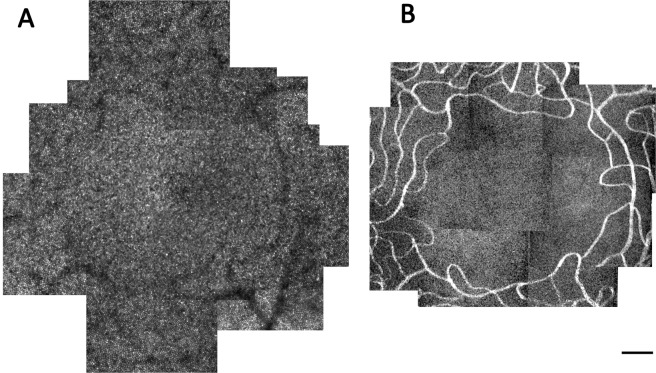
(**A**) Representative AOSLO cone photoreceptor mosaic acquired over the central 4° of the fovea in a 6 year-old child. (**B**) Capillary perfusion image surrounding the foveal avascular zone for the same subject at the same spatial scale as in (**A**). Scale bar = 100 μm.

Montages of the cone mosaic were generated by manually stitching individual confocal registered images using Adobe Photoshop (Adobe Systems Inc., San Jose, CA USA). A representative example of a montage of the cone mosaic from a 6-year old child is shown in Fig. 6A. Because it was not possible to resolve the central-most foveal cones in all subjects to identify the location of peak cone density (i.e., the location with a retinal eccentricity of 0°), we used a technique developed by Putnam *et al*.^47^ to estimate the location of peak cone density. In brief, a custom MATLAB program was used to semi-automatically mark all resolved cones within the central 3° of the fovea^48^ and generate a topography map of cone density values. Separate ellipses were then fit to locations in the foveal mosaic that contained 65–70%, 71–75%, and 76–85% of the maximum cone density measured in the montage. The centers of these 3 ellipses were averaged to estimate the location of peak cone density.

A custom, semi-automated program (Mosaic Analytics, Translational Imaging Innovations, Hickory, NC USA)^49^ was used to calculate cone metrics within a 37 × 37 μm sampling window at eccentricities of 0.2 mm, 0.3 mm, and 0.5 mm from the foveal center along all 4 major meridians. Linear and angular bound cone densities (cones/mm^2^ and cones/deg^2^, respectively) were calculated as the ratio of the number of bound Voronoi cells to the summed area of the bound Voronoi cells^48,50^. Farthest neighbor distance was calculated as our spacing metric, as Cooper *et al*.^49^ found this metric to be most sensitive to a change in the total number of cones marked within an image out of the four metrics that were examined (including density recovery profile distance, nearest neighbor distance, farthest neighbor distance, and intercell distance). Farthest neighbor distance was calculated as the distance (in μm) between a given cone and its farthest neighbor, where the neighbors of a given cone are comprised of all cones within adjacent Voronoi cells. Cone metrics were averaged across all 4 meridians for analyses performed across all eyes (regardless of refractive error). For analyses examining the impact of different meridians on cone density and spacing, metrics were averaged along the horizontal meridian (nasal, temporal) and vertical meridian (superior, inferior) separately.

AOSLO videos of inner retinal vasculature were acquired by shifting the focal plane anteriorly to a plane where the capillaries were in best focus. After correcting for scan distortions and stabilizing videos, perfusion images were created using a method similar to that employed by Chui *et al*.^37^. This technique is based on the premise that the largest changes of intensity (or greatest standard errors) occur over time in regions of the video where blood is actively flowing as opposed to retinal regions that are more static in their structure (e.g., devoid of perfused vasculature). Motion-corrected videos were normalized to the maximum intensity value of any pixel in the video and were median filtered using a 3 × 3-pixel kernel to reduce noise. Each 150- to 250-frame filtered video was divided into 25-frame intervals in order to limit the influence of slower (less than 0.5 Hz) tissue reflectance changes on the perfusion image. The standard-error of each pixel over the 25-frames was computed as the standard error frame, which was subsequently median filtered by a 3 × 3-pixel kernel. All available filtered standard-error frames were averaged to produce a standard-error image. This image was then normalized to its maximum value and histogram-stretched so that the lower and upper 1% of the histogram were set to 0 and 255, respectively.

Registered perfusion images were stitched together using Adobe Photoshop to create a montage of the perfused FAZ (Fig. 6B). The border of the FAZ was semi-automatically traced two times using the NeuronJ plug-in for ImageJ^51^. The coordinates of the border were then imported into in a custom MATLAB program to quantify FAZ metrics. The area of the FAZ was calculated by multiplying the area of one pixel (in mm^2^) by the number of pixels enclosed by the border of FAZ. The perimeter of the FAZ was calculated as the total length of the FAZ border (in mm). Axis ratio was calculated as the ratio of the length of the major axis to that of the minor axis of an ellipse best-fit to the border of the FAZ. Effective diameter (in µm) was defined as the diameter of a circle with an area equivalent to the calculated FAZ area. Circularity was calculated as:

3 $${Circularity}=4\pi \left(\frac{{area}}{{{perimeter}}^{2}}\right)$$

where a value of 1 indicates a perfect circle and a value closer to 0 indicates a polygon shape. Acircularity index measures the degree of irregularity in the overall shape of the FAZ and was calculated as the ratio of the FAZ perimeter to the perimeter of a circle with an area that is equal to the FAZ area^52^. A perfectly circular FAZ has an acircularity index of 1. The value of the acircularity index increases with increasing deviation of the shape of the FAZ from a circle.

### Statistical analysis

Data are presented as mean ± standard deviation. Multiple linear regression models were conducted to examine the relationships of cone density, cone spacing, FAZ size, and foveal pit metrics with age and axial length across all eyes. To reduce the false-discovery rate associated with multiple statistical testing, a Bonferroni correction was applied and a *P* value of <0.0026 was considered significant. To compare FAZ and foveal pit metrics between myopic and non-myopic children, unpaired t tests (or Mann-Whitney Rank Sum tests) were used for metrics following a normal (or non-normal, as determined by Kolmogorov–Smirnov test) distribution. A three-way ANOVA was performed for the analysis of cone density and spacing to study interactions between refractive error, eccentricity and meridian. A *P* value <0.05 was considered statistically significant.

## Data Availability

The datasets generated and analyzed during the current study are available from the corresponding author upon request.
